# Endoscopic removal of a retained surgical sponge in a young Syrian refugee after Caesarean section: a case report with discussion of cultural and political consequences

**DOI:** 10.1186/s13037-016-0111-z

**Published:** 2016-10-26

**Authors:** Johannes Ackermann, Moritz Kanzow, Micaela Mathiak, Ulrich Pecks, Nicolai Maass, Ibrahim Alkatout

**Affiliations:** 1 Department of Gynecology and Obstetrics, University Hospitals Schleswig-Holstein, Campus Kiel, Arnold-Heller-Str. 3, Haus 24, 24105 Kiel, Germany; 2 Institute of Pathology, University Hospital Schleswig-Holstein, Campus Kiel, 24105 Kiel, Germany

**Keywords:** Retained instrument, Retained sponge, Language barrier, Refugee, Safety strategies, Complications, Diagnostics, Laparoscopy

## Abstract

**Background:**

Inadvertently retained sponges and instruments still constitute a major but preventable complication in surgery. Given the high geographic mobility of patients, the fluctuation of physician-patient contact, and communication problems due to language barriers, the conscientious use of structured safety protocols in clinical routine is an essential aspect of quality in health care.

**Case presentation:**

We report the case of a 24-year-old refugee from Syria who presented at our gynecological outpatient department with a tumor in the lower abdomen, suspected to be a lump in the ovary or the uterus. Language barriers hindered exact recording of the patient’s medical history. We knew she had undergone three Caesarean sections several years ago. The diagnostic laparoscopy unexpectedly revealed a tumor suspected to be a retained surgical sponge. The lesion was removed completely and the patient discharged from the clinic five days later.

**Conclusion:**

In ambiguous cases, the diagnostic and therapeutic potential of minimally invasive surgery ensures safe and effective treatment of the patient, a short hospital stay, and low rates of complications. Especially in cases of language and/or cultural barriers, structured safety protocols should be a part of clinical routine in order to prevent unnecessary complications.

## Background

Inadvertently retained sponges and instruments constitute a major but preventable complication in surgery [1]. Risk factors for a retained foreign object during surgery include errors in counting or recording the sponges, a high body mass index, an unexpected change in the surgical procedure, emergency surgery, and a change in personnel during the operation [2]. The incidence of retained surgical instruments varies from 1 in every 1500 elective surgeries to 1 in every 300 to 700 emergency surgeries [3, 4]. In a current study up to 43 % of surgeons reported that they had already left foreign bodies in a patient after a surgical procedure and 73 % asserted the removal of one or more foreign bodies [5]. Surgical sponges are the most common foreign body retained inadvertently during surgery because of their frequent use, their varying sizes, and their loss of initial appearance when saturated with blood [3, 4]. The clinical presentation of retained surgical sponges and the time taken to detect their presence are highly variable [6]. The condition may be accompanied by massive abdominal pain and be diagnosed within a few hours, or the patient may have no symptoms for decades. In the latter case the emergence of symptoms depends mainly on the reaction of the immune system to the foreign body (Table 1) [4, 6].

**Table 1:** Symptoms and differential diagnosis of inadvertently retained sponges and instruments

Symptoms	Clinical appearance of inadvertently retained sponges and instruments	Differential diagnosis
Infection	Infection at the surgical site with fever, pain and sepsis	Wound infection of other origin, pneumonia, infection of the catheter, urinary tract infection
Acute pain	Acute pain, becoming more extensive, often accompanied by fever and infection	Wound pain, postoperative hemorrhage
Chronic pain	Chronic pain persisting after the intervention without any other correlate	Adhesions, nerve damage
Tumor	Unspecific tumor mass around the surgical site	Coagulum, tumor of other origin, adhesions
Fistulization	Fistulization with suspected material of no natural origin	Fistulization because of disturbed wound healing, infection, or fistulization due to other causes
Obstruction	Obstruction because of fistulization or swelling of the retained object	Tumor of other origin, adhesions
Hemorrhage	Gastrointestinal, vaginal, or urinary hemorrhage because of fistulization	Ulcer, tumor

Prevention of retained surgical sponges and instruments is a crucial step in quality and safety management. Prevention strategies include standardized counting protocols, radiographic screening, counting devices and detection devices. Sponges or instruments tend to be left behind despite the fact that counting protocols are a part of nearly all surgical procedures. In as many as 88 % of cases of retained foreign surgical objects, the sponge and instrument count was reported to be correct [1, 7–9].

We report the case of a 24-year-old refugee from an Arabian country who presented at our gynecological outpatient department with a tumor in the lower abdomen that looked like a retained surgical sponge on explorative laparoscopy. We discuss prevention strategies for such incidents, especially in current times of geographic mobility, fluctuation of physician-patient contact, and growing communication problems due to language barriers, given the large numbers of refugees and immigrants in Western countries.

## Case presentation

A 24-year-old woman presented at our gynecological outpatient department with a tumor in the lower abdomen, suspected to be a lump in the ovary or the uterus. The tumor was discovered by her gynecologist at a routine transvaginal ultrasound investigation. The woman was a refugee from Syria. Communication was hindered by language differences and our inability to obtain an adequate translator.

Her medical history included pain, fever, weight loss, and menstrual disorders. She had undergone three Caesarean sections, the last of which had been performed two years ago in a European country. Further details of her medical history were not known.

**Fig. 1:**
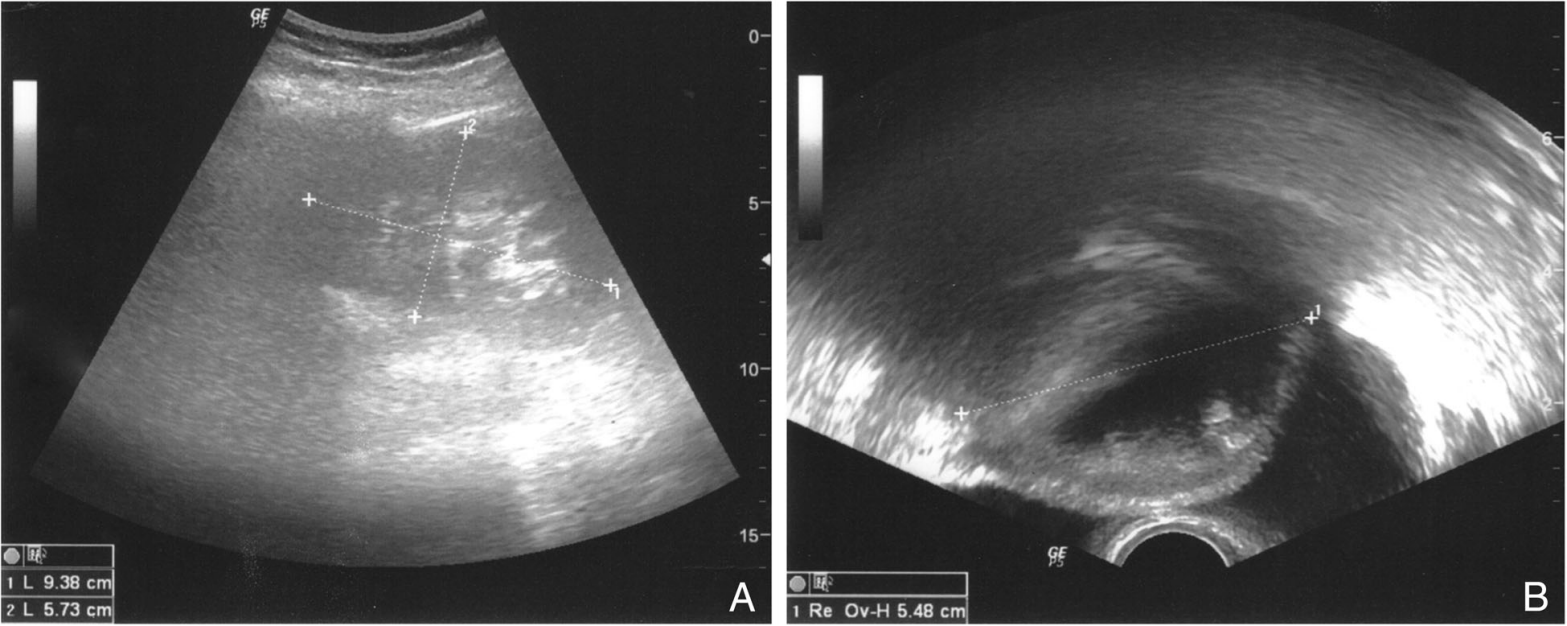
**a** Transabdominal ultrasound. **b** Transvaginal ultrasound shows the suspected tumor in the lower abdomen, measuring about 5.5 × 5.7 × 9.4 cm. The cyst has a solid as well as fluid content and an anechoic area behind the cyst. The bladder is not involved

The gynecological examination showed a painless palpable resistance to the right of the uterus. Our transvaginal and transabdominal ultrasound investigation revealed a cystic lesion measuring 9.4 cm × 5.7 cm in this area (Fig. 1). We suspected the tumor to be a malignancy of the ovary or a pseudocystic lesion as a consequence of the three Caesarean sections. No further imaging diagnostics were done. Clinical chemistry showed no sign of infection or malignancy, such as an increasing CRP or tumor markers (CA 125).

**Fig. 2:**
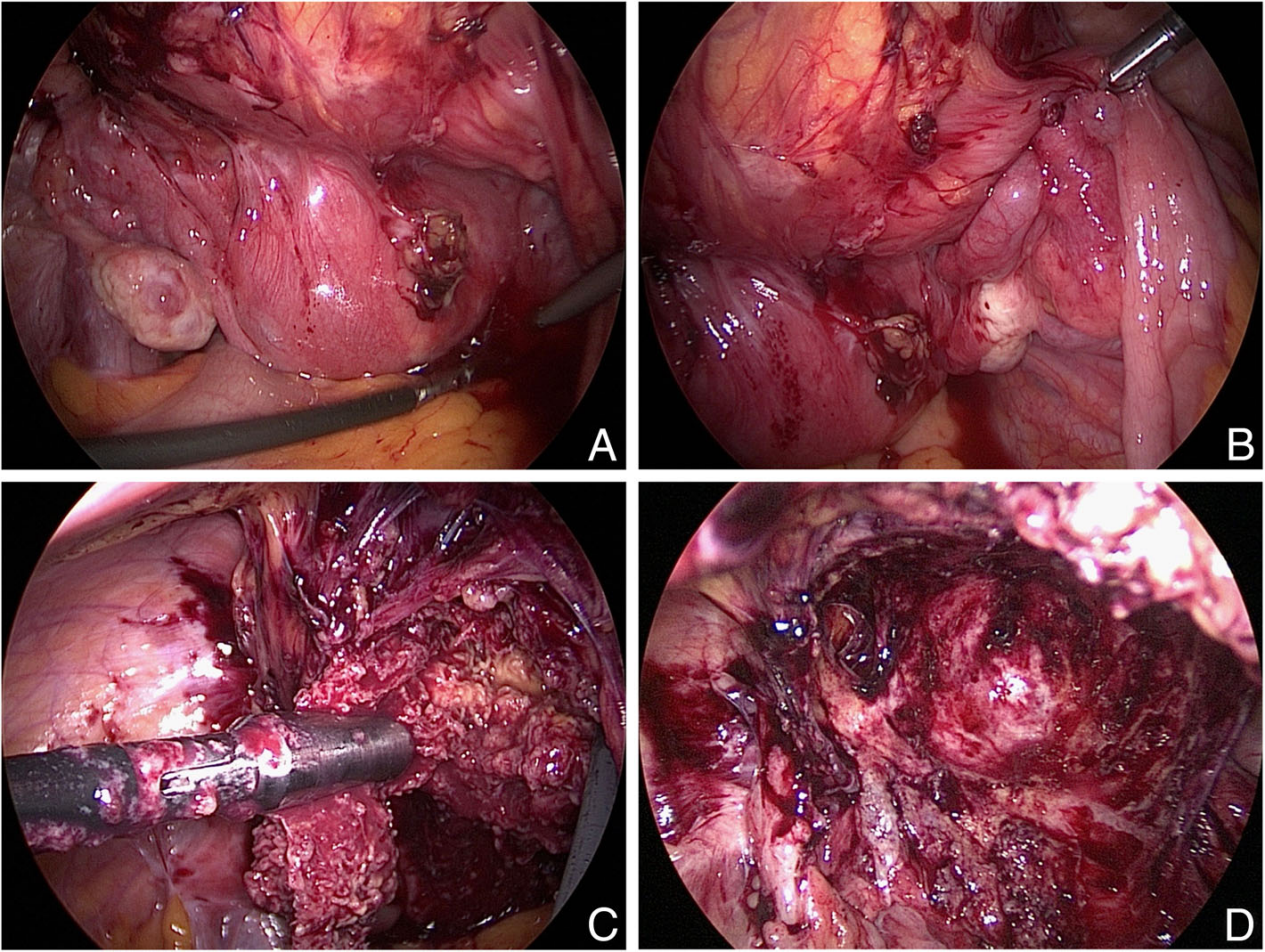
Laparoscopic view of the patient’s lower abdomen. **a** + **b** Tumor mass between the uterus and the bladder. Both ovaries are free of suspected lesions. **c** The cyst was opened to remove the infectious fluid and revealed a solid foreign mass. The surgical sponge is removed laparoscopically. **d** The surgical site after complete removal of the sponge. The intact bladder is seen in the deeper aspect, after retrograde blue dye filling. The complete capsule of the tumor was removed and bleeding was observed in the wound bed

We decided to perform a laparoscopic exploration and remove the tumor either by laparoscopy or laparotomy. In accordance with clinical findings, the operation site showed a swelling between the uterus and the bladder (Fig. 2). The ovaries and other abdominal sites were free of lesions. We decided to remove the tumor by laparoscopy, which then turned out to be a retained surgical sponge with granulation tissue (Fig. 2). The foreign body and the surrounding tissue were removed completely.

The patient was given preventive intravenous antibiotic treatment with cefuroxim and metronidazol postoperatively for five days, based on the likelihood of infection of the surgical sponge. After completion of antibiotic treatment she was discharged from the hospital on the sixth day, with no symptoms or restrictions.

**Fig. 3:**
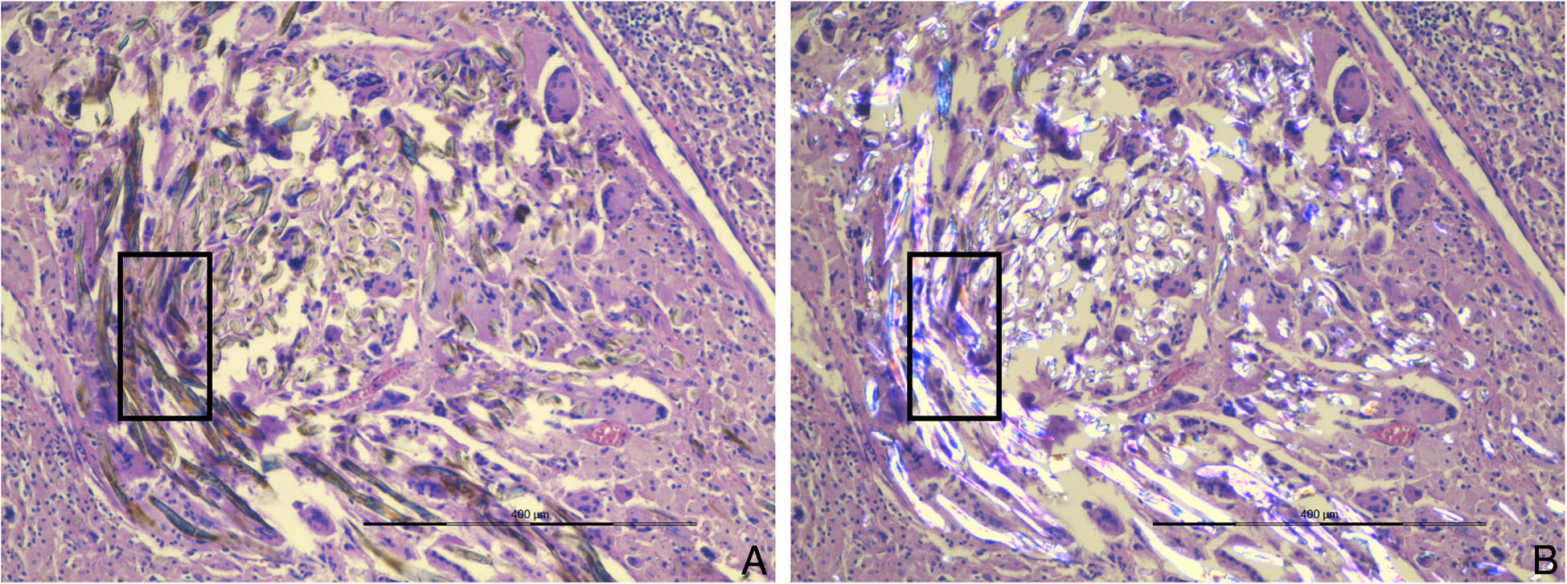
Histological work-up of the retrieved material (scale 400 μm). In addition to vascularized fat and connective tissue we found (**a**) dense fibroblast proliferation with extensive macrophage clusters and multinuclear giant cells (box), which have phagocytosed filiform foreign matter that turns birefringent in polarized light (**b**)

The diagnosis of a retained surgical sponge was verified by histological examination (Fig. 3).

## Discussion

**Fig. 4:**
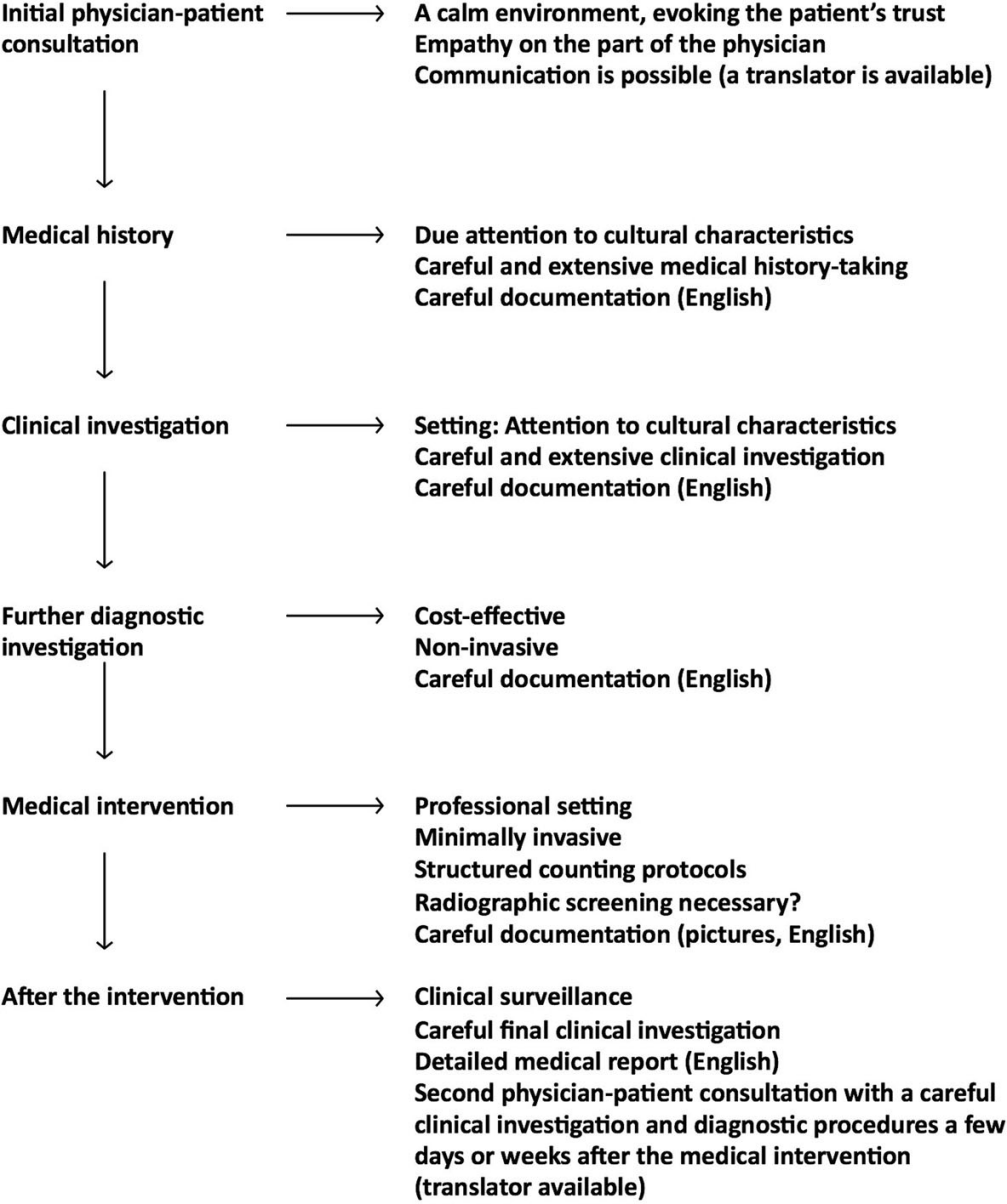
Structured safety protocol for clinical routine. The protocol can be used at every physician-patient consultation, but is also modified for patients from other countries with different cultures and/or communication problems due to language barriers

Since the patient’s welfare is of prime importance in any medical intervention [10], safety strategies play a crucial role in all medical procedures. Especially in times of high geographic mobility, fluctuation of physician-patient contact, and communication problems due to language barriers, the conscientious use of structured safety protocols in clinical routine is an essential aspect of quality in health care (Fig. 4).

Inadvertently retained sponges and instruments constitute a rare medical complication. When it does happen, the event may cause severe harm to the patient as well as professional and medico-legal consequences for the physician and the hospital [11]. In a current study up to 43 % of surgeons reported that they had already left foreign bodies in a patient after a surgical procedure and 73 % asserted the removal of one or more foreign bodies. For the patient it may result in morbidity, acute or chronic pain, infection, misdiagnosis, and several subsequent operations [11]. Often forgotten and underrated are the psychological, emotional, and financial problems for the patient. Consequences for the physician or the hospital include the costs of subsequent treatment, compensation, legal proceedings, a negative public image, and loss of confidence on the part of patients.

Prevention strategies should be used in any medical intervention in order to avoid these grave consequences. This includes structured counting protocols, radiographic screening, counting devices, and detection devices [11].

Structured counting protocols are the easiest means of preventing retention of a foreign body and should be a part of any medical intervention. A standardized counting protocol should include an initial count before the start of the procedure, a count before closure of a cavity within a cavity, a count when wound closure starts, and a final count at the end of the procedure [12]. Nevertheless, since counting is done by individuals, human error is possible. This is the reason why inadvertently retained sponges or instruments are found in as many as 88 % of operations in which the sponge and instrument count was declared to be correct [1]. Reliable prevention and detection procedures are needed to avoid such human error, especially in operations involving a high risk for a retained sponge or instrument. These high risk situations include emergency surgeries, unexpected changes in surgical procedures or personnel, and patients with a high body mass index [2].

Programs that included educating the perioperative staff members, standardizing count practices, formally reviewing every reported count discrepancy with the nursing team, and reviewing and revising the count policy for prevention of retained surgical items could show a reduction of the number of incorrect counts and count discrepancies by 50 % [13].

Radiographic screening is a tested additional procedure for such prevention and detection [3]. It should be performed whenever counting is declared incomplete. Routine radiographic screening protocols after surgery have been tested in the clinical setting, and were found to be highly sensitive in detecting retained sponges and instruments. The disadvantages of routine screening include the need for high-resolution survey radiographs, high costs, and radiation exposure [3]. Other strategies such as counting and detection devices have been tested, but not incorporated into clinical routine because of their cost as well as the time and the effort involved. Nevertheless, they are promising methods to enhance patient safety and welfare [11]. Future investigations will show whether these systems can be integrated into clinical routine.

## Conclusions

The patient we reported on in this article is a refugee from Syria who had travelled through many countries on her way to Germany. The last Caesarean section was performed in a developed country. We had no information about the operation and the potential complications that could have led to the retained sponge. We performed diagnostic laparoscopy because of a suspected malignancy of the ovary or pseudocystic lesions as a consequence of the preceding Caesarean sections. No further imaging diagnostics were carried out because laparoscopy represents an outstanding diagnostic and therapeutic tool in cases of uncertain gynecological lesions. Nevertheless, other imaging procedures, such as computer tomography or magnetic resonance imaging, can help to diagnose a retained surgical sponge. Especially in this case, complicated by language barrier and an inadequate history, laparoscopy offered a safe, effective treatment with a shorter hospital stay. Given the mobility of patients in current times and the fact that many are lost to follow-up, diagnostic and therapeutic procedures must provide maximum safety and ensure the patient’s welfare to the greatest possible extent.
